# A Phase 2, Randomized, Double-Blind, Placebo-Controlled Trial of CX-8998, a Selective Modulator of the T-Type Calcium Channel in Inadequately Treated Moderate to Severe Essential Tremor: T-CALM Study Design and Methodology for Efficacy Endpoint and Digital Biomarker Selection

**DOI:** 10.3389/fneur.2019.00597

**Published:** 2019-06-11

**Authors:** Spyros Papapetropoulos, Margaret S. Lee, Stacey Boyer, Evan J. Newbold

**Affiliations:** ^1^Cavion Inc., Cambridge, MA, United States; ^2^Cavion Inc., Charlottesville, VA, United States

**Keywords:** essential tremor, CX-8998, T-type calcium channel modulator, proof of concept, TETRAS

## Abstract

**Background:** Essential tremor (ET) is a common, progressive neurological syndrome with bilateral upper-limb dysfunction of at least 3-year duration, with or without tremor in other body locations. This disorder has a negative impact on daily function and quality of life. A single oral therapy has been approved by FDA for ET. Off-label pharmacotherapies have inadequate efficacy and poor tolerability with high rates of patient dissatisfaction and discontinuation. Safe and efficacious pharmacotherapies are urgently needed to decrease tremor and improve daily living. T-CALM (Tremor-CAv3 modulation) protocol is designed to assess safety and efficacy of CX-8998, a selective modulator of the T-type calcium channel, for ET therapy.

**Methods/Design:** T-CALM is a phase 2, proof of concept, randomized, double-blind, placebo-controlled trial. Titrated doses of CX-8998 to 10 mg BID or placebo will be administered for 28 days to moderate to severe ET patients who are inadequately treated with existing therapies. The primary endpoint will be change from baseline to day 28 of The Essential Tremor Rating Assessment Performance Subscale (TETRAS-PS). Secondary efficacy endpoints for clinician and patient perception of daily function will include TETRAS Activity of Daily Living (ADL), Quality of Life in Essential Tremor Questionnaire (QUEST), Clinical Global Impression-Improvement (CGI-I), Patient Global Impression of Change (PGIC), and Goal Attainment Scale (GAS). Kinesia One, Kinesia 360, and iMotor will biometrically evaluate motor function and tremor amplitude. Safety will be assessed by adverse events, physical and neurological exams and laboratory tests. Sample size of 43 patients per group is estimated to have 90% power to detect a 5.5-point difference between CX-8998 and placebo for TETRAS-PS. Efficacy analyses will be performed with covariance (ANCOVA) and 2-sided test at 0.05 significance level.

**Discussion:** T-CALM has a unique design with physician rating scales, patient-focused questionnaires and scales and objective motor measurements to assess clinically meaningful and congruent efficacy. Patient perception of ET debilitation and therapy with CX-8998 will be key findings. Overall goal of T-CALM is generation of safety and efficacy data to support a go, no-go decision to further develop CX-8998 for ET. Design of T-CALM may guide future clinical studies of ET pharmacotherapies.

**Clinical Trial Registration:** www.ClinicalTrials.gov, identifier: NCT03101241

## Introduction

### Background

Essential tremor (ET) is described as a progressive neurological disorder that elicits involuntary rhythmic trembling of the hands, head, larynx, legs or trunk. ET is considered a syndrome because it is not a single disease and has several possible etiologies (1). A task force of The International Parkinson's Disease and Movement Disorder Society has recently proposed a more succinct definition of ET. This society defined ET as a syndrome with isolated bilateral upper-limb action tremor of at least a 3-year duration, with or without tremor in other body locations (e.g., head, lower limbs). It was also pointed out that other neurologic symptoms, such as dystonia and Parkinsonism, are not associated with ET (2). ET is often inherited as autosomal dominant but tremor-inducing drugs and toxins are also implicated in the causality of the syndrome (3). The pathophysiology of ET is being investigated but several studies have identified multiple abnormally oscillating neuronal circuits connecting the cerebellum, inferior olive, thalamus and areas of the cortex as etiologies (1, 3, 4). T-type calcium channels (TTCC) are reported to regulate the inferior-olive-cerebellum and thalamocortical neuronal networks. Increased activation of these channels has been shown to promote excessive rhythmicity in these neuronal networks and has been identified as a key pathophysiology and potential therapeutic target for ET (5–9).

To meticulously evaluate the presence of ET, a comprehensive medical history and neurologic examination are recommended. Age of onset, family history, timing of progression, use of tremor-inducing drugs, and exposure to toxins are critical components of the medical history (1, 4). The neurologic examination should include the body locations of tremor and arousal conditions for tremor such as rest, posture, and purposeful movements (1, 4). Tremor rating scales are also employed to assess severity, degree of disability, quality of life, and effect on activities of daily living (10). The Essential Tremor Rating Assessment scale (TETRAS) was developed by The Tremor Research Group to replace older instruments such as the Fahn-Tolosa-Marin (FTM) scale and to improve psychometric properties, dynamic range, expediency, accuracy, and comprehensive quantification of ET severity (11). Electrophysiology techniques, such as electromyography and accelerometry, have also been utilized for differential diagnosis of ET (12).

ET is reported to be a widespread movement disorder with a 1% incidence worldwide (13). Population-based incidence studies of ET with U.S. census data of 2012 revealed that 2.2% of the U.S. displayed ET (14). It is estimated that there may be more than 7 million ET patients in the U.S. (14). The frequency of ET is directly correlated with increase in age and generally comparable in males and females (1, 13). It is estimated that 8 times as many people have ET compared to Parkinson's Disease (14). As the diagnostic tools for ET become more sophisticated, the prevalence of the disorder may be amplified. ET has been detected as early as childhood with incidence peaks in the second and sixth decades (15).

Although ET does not seem to adversely affect life expectancy, the syndrome has a major effect on a patient's ability to adequately function on a daily basis. Activities at home and at work are disrupted and quality of life and social networking are compromised (16, 17). ET is recognized as a multisymptomatic disturbance that impacts writing, dressing, eating, self-care, mood, memory, attention, communication, and sleep and is a source of anxiety, depression, and social isolation (18–20). Disability is reported in more than 90% of ET patients who seek medical care and may be significant enough to warrant invasive surgery such as deep brain stimulation (DBS) or thalamotomy (21). Severely affected patients are unable to feed or dress themselves (22). Due to uncontrollable shaking, 60% of ET patients choose not to apply for job promotions and 15–25% are forced to retire prematurely (15). In addition to ET, patients may also have intention tremor (23), rest tremor (24), and other motor abnormalities, including ataxia (25). This diverse group of tremor types is disabling and causes functional limitations (21, 26). Resting and intention tremors are associated with illness of increased duration in ET patients (21, 24, 27) and suggest that the complexity of tremor phenomenology and severity of ET progressively increase with longstanding disease. ET cases with resting tremors had disease duration of 32.1 ± 24.5 years compared to those without resting tremors (19.6 ± 16.8 years) (24). Higher overall tremor scores were reported in ET patients with intention tremor (23). Disease duration correlated with intention tremor severity. Although frequency of ET tremors generally decreases over time, the amplitude of the tremors gradually increases (28).

ET is a common movement disorder with a major unmet medical need. Therapy for ET consists of drugs that often provide limited efficacy and/or poor tolerability. Neurosurgical interventions (DBS, gamma knife and focused ultrasound thalamotomy) are generally effective with acceptable tolerability but they are invasive and restricted to the most severe (1–3%) ET patients (1, 29, 30). There are a limited number of safe and efficacious pharmacotherapies for ET. Inconsistent data from clinical trials with small numbers of ET patients are partially responsible for the paucity of approved pharmacotherapies. Published ET clinical trials have been hampered by uncertain diagnosis of ET patients, cross-over design with carryover effect, small numbers of patients, open-label, diverse controls, limited methods for assessment of tremor amplitude reduction, and short duration of treatment (1, 31). Most of the available pharmacotherapies treat the symptoms rather than the cause of ET and were originally developed and approved for other indications (1, 29, 31). Propranolol, a non-selective β-adrenergic receptor antagonist, is the only FDA-approved (1967) pharmacologic agent for ET. This approval in 1967 was based upon a 2 week, randomized, double-blind, parallel, placebo-controlled trial with only 9 ET or familial tremor patients (32). At doses of 40–80 mg three times daily (TID), propranolol reduced tremor severity compared to placebo in this study of a small number of patients. More recent clinical trials of propranolol as a monotherapy in drug-naïve ET patients have confirmed that the response rate is 50 to 70% with an average tremor diminution of 50% compared to placebo (4). Propranolol has not generally been effective in patients with severe ET. Based upon clinical evidence, ET pharmacotherapies have been designated as first, second and third line agents (4). Propranolol and primidone are considered first line because they have been evaluated for safety and efficacy in randomized clinical trials of ET with class 1 evidence. Gabapentin, pregabalin, topiramate, clonazepam, alprazolam, and metoprolol are considered second line agents due to a dearth of class 1 evidence from randomized ET clinical trials. On the basis of open label or case studies, nimodopine and clozapine are classified as third line treatments. Due to a limited timeframe and low rate of effectiveness for tremor-induced physical and mental impairments and intolerable side effects, the three lines of ET pharmacotherapies have a high percentage of patient dissatisfaction and discontinuation (1, 33–35). Thus, in addition to standard of care, there is a definitive need for novel, durably effective ET pharmacotherapies with minimal side effects that have a beneficial impact on not only amplitude of tremor but also activities of daily living and other functional outcomes. This goal can be achieved with additional research for more clinically meaningful therapeutic targets and a better understanding of ET pathophysiology and clinical and genetic heterogeneity.

TTCC have been shown to function as low threshold, voltage-gated calcium channels and are located primarily in neurons (36). TTCC activate upon weak depolarization of the neuronal cell membrane and permit calcium entry into excitable cells at the onset of action potential. Under abnormal states, the TTCC CAv3 subtype is upregulated or has increased activity that makes it a prime target for neurologic disorders such as ET (37–39). CAv3 isoforms are expressed in neurons throughout the central and peripheral nervous systems. CAv3 has been shown to be a mediator of subthreshold oscillations and excessive rhythmicity in neurologic disorders such as tremor, neuropathic pain, epilepsy, and Parkinson's disease (7, 40–42). The inferior olive has been reported to function as the inducer and intrinsic pacemaker of tremor in animal models (43). CAv3 is highly expressed in the IO and cerebellum. Tremor-related oscillations in the olivocerebellar pathway are key abnormalities underlying ET and trigger onset of tremor-related rhythms (38). Harmaline, a plant alkaloid that effects the cerebellum and IO, induces tremor in animals. Harmaline-induced tremor in animals is comparable to some of the clinical manifestations of human ET and is used as an experimental model for evaluation of efficacy of pharmacotherapies (40, 44).

Compounds that target TTCC have been studied for their beneficial effects on ET. Clinical studies of zonisamide and topiramate, two drugs with nonspecific TTCC inhibitory activity, have demonstrated effectiveness for ET. However, unacceptable side effects that result in premature discontinuation have limited further development of these agents for ET (1, 6, 45, 46). CX-8998, a potent, highly selective and state dependent small molecule modulator of TTCC, is under development for treatment of ET (47). Robust efficacy of CX-8998 (and analogous TTCC modulators) has been shown in numerous rat models of CNS disorders including tremor, generalized epilepsy, neuropathic pain, psychosis, and insomnia (48–51). Several unpublished, nonclinical safety pharmacology studies have documented selectivity, biologic activity and a wide safety margin of CX-8998. Four phase 1 single and multidose safety studies in healthy volunteers, a phase 2A trial in acute psychosis in schizophrenics (52) and clinical pharmacokinetic and pharmacodynamic studies have been conducted. The data from these CX-8998 clinical studies (200 plus patients) have shown that single doses up to 18 mg and multiple doses from 2 to 12 mg for 7 days were well tolerated with transient and mild to moderate AEs. In the phase 2A trial of acute psychosis, 8 mg twice daily (BID) was generally well tolerated. Clinically relevant patterns of abnormalities were not detected in blood and urine laboratory tests, electrocardiograms, physical examinations or pulmonary function in any of the clinical studies. Based upon the favorable safety data from these nonclinical and early clinical studies, CX-8998 was selected for a phase 2 proof of concept study to assess its safety and efficacy for reduction of the severity of ET at doses up to 10 mg twice a day for 4 weeks.

### Protocol Outline and Specific Aims

CX-8998 will be evaluated for safety and efficacy in a phase 2, multicenter, randomized, double-blind, placebo-controlled, parallel-group, proof of concept trial in patients with moderate to severe ET. The overall goal of the T-CALM (Tremor-CAv3 modulation) trial is to provide safety and efficacy data that will support a positive or negative decision for late stage clinical development of CX-8998 for treatment of ET. Other goals are to demonstrate efficacy of CX-8998 through relevant and converging endpoints, evaluate performance scales and objective biometric methodologies underlying ET efficacy endpoints, and generate a better clinical understanding and definition of ET patients through clinically relevant trial selection criteria. Plasma exposures of CX-8998 and metabolites will also be evaluated.

If the T-CALM proof of concept trial demonstrates that CX-8998 is effective with a favorable safety and tolerability profile, late stage clinical development will be undertaken and potentially support regulatory approval as a novel, selective, durable, and potent ET pharmacotherapy. The T-CALM study may also generate meaningful guidelines for design, patient selection, and relevant and convergent efficacy endpoints for future clinical trials of novel ET pharmacotherapies. The primary aim of the main T-CALM study is to assess the efficacy of CX-8998, at doses up to 10 mg BID, for reduction of severity (amplitude) of ET. An optional additional component of the main T-CALM study is the T-CALM digital substudy that will evaluate the feasibility of three different digital monitoring platforms for accurate quantification of changes in motor function in ET patients.

## Stepwise Procedures AND Endpoints

**Figure 1:**
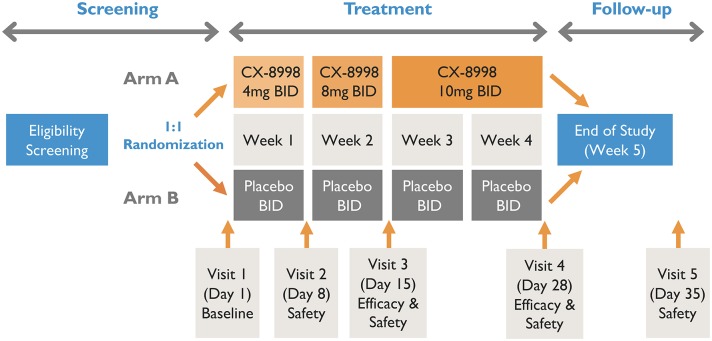
Schematic of T-CALM Design.

A schematic of T-CALM study design is presented in Figure 1.

The main T-CALM study is designed as a proof of concept, multicenter, double-blind, randomized, placebo-controlled, parallel-group trial. The screening period will be up to 4 weeks. Before any study procedures are conducted, patients will read and sign the IRB-approved informed consent in the presence of the investigator or suitable designee. Primidone (a strong CYP3A4 inducer) use is excluded due to the potential of CX-8998 to be subject to CYP3A metabolism. Thus, patients taking primidone will be given 6 weeks of screening to allow for safe discontinuation of the drug. Stable doses of a single anti-tremor medication other than primidone as a standard of care will be allowed during the study.

The study will enroll a population of moderate to severe ET patients inadequately treated with standard of care approaches at 22 clinical sites in the U.S. The clinical sites are identified online at ClinicalTrials.gov under registration number NCT03101241.

Key eligibility criteria for the main T-CALM study are as follows:

1. Signed, informed consent will be obtained for all study participants.
2. Males and females 18–75 years of age will be enrolled.
3. Patients with moderate to severe ET and initial diagnosis prior to age 65 will be included.
4. Tremor severity score of at least 2 in at least one upper limb of the 3 maneuvers on TETRAS-PS.
5. TETRAS-PS score of at least 15 at screen.
6. On stable doses of up to one concurrent anti-tremor medication permitted; use of strong CYP inducer primidone will be excluded.
7. Surgical intervention excluded.

A complete list of inclusion/exclusion criteria for the main T-CALM study is available online at ClinicalTrials.gov under registration number NCT03101241.

Eligibility criteria for the optional T-CALM digital substudy are as follows:

1. Patients must meet all eligibility criteria of the main T-CALM study protocol.
2. Patients must be able to comply with user requirements as assessed by site personnel.
3. Patients will not be issued digital devices/downloads until they have consented to participate in the optional T-CALM digital substudy.

Patients will be randomized in a 1:1 ratio to receive CX-8998 or placebo with an interactive web response system (IWRS). Randomization will be stratified by concomitant use of anti-tremor medication and by type of site (main study vs. substudy). The randomization code will be prepared by an unblinded statistician who is uninvolved in conduct of the study. Patients that meet screening criteria will be randomized to treatment group A or B. Group A will receive titrated doses of CX-8998 up to 10 mg BID. Group B will receive matching placebo. Randomized study participants will enter a 4-week, double-blind, dose titration period followed by a 1-week safety follow up after the last dose of study medication. At baseline (day 1), patients will have safety and tremor evaluations prior to administration of study treatments. During the first week, patients will receive 4 mg of study drug or matching placebo twice daily (BID). On day 8 (week 2), patients will be assessed at the clinic for safety and dose titration to 8 mg (or matching placebo) BID. On day 15 (week 3), patients will report to clinic for safety and efficacy evaluations and final dose titration to 10 mg (or matching placebo) BID. The final efficacy visit will be day 28 (week 4). The final safety visit will take place on day 35 (week 5). Blood samples will be collected pre-dose on days 8, 15, and 28 and at approximately 4 h post-dose on day 28 for plasma concentration measurements of CX-8998. If intolerable adverse events (AEs) are evident at any of the doses, the dose may be decreased to the next lowest dose at day 8 or 15 or at any time prior to those scheduled visits. If the lowest scheduled dose (4 mg BID) is intolerable, it can be decreased to 2 mg BID. After dose reduction, an increase in dose will not be allowed. If patients do not tolerate dose reduction, they will be withdrawn from treatment.

Patients that have been screened and met the eligibility criteria for the main T-CALM study will have the option to additionally participate in the T-CALM digital substudy. Enrollment of patients in the substudy will be randomized to CX-8998 or placebo in the same proportion (1:1) as the main study. After informed consent is signed, patients will be given the option to use one or both of two digital tools, iMotor or Kinesia 360, or to conduct additional testing with Kinesia One for objective measurement of motor function. The dosing regimen and schedule of safety assessments for the substudy will be identical to that of the main study. At the screening visit, patients in the iMotor arm of the substudy will have evaluations completed in the presence of a study staff member. Patients in the Kinesia 360 arm of the substudy will wear the device to collect data for 2 days after screening and then return the device to the study site. Data collected for these 2 days will serve as baseline motor assessments for Kinesia 360. At baseline (day 1), baseline iMotor evaluations will be taken prior to dosing and after completion of the main T-CALM safety and tremor assessments. At the end of weeks 1 and 2, safety and Kinesia 360 and iMotor evaluations will be collected. The final efficacy measurements for both devices will be collected at the end of week 4 visit. The final safety visit will occur at the end of week 5.

The sponsor, patients, investigators, and any others involved in conduct of the study or analysis of data will be unaware of treatment assignments until the study is unblinded. An individual patient's treatment assignment will be unblinded only when knowledge of the treatment is necessary for medical management of the patient or if required for reportable safety events such as an unexpected serious adverse reaction. CX-8998 is formulated in size 4 hard gelatin capsules with 2 mg of hydrochloride salt of the active pharmaceutical ingredient mixed with a blend of excipients. Matched placebo is formulated in size 4 hard gelatin capsules with a blend of comparable excipients.

The primary, secondary, and exploratory efficacy endpoints for the main T-CALM study are listed below:

The primary endpoint is the change from baseline to day 28 of the TETRAS performance subscale (TETRAS-PS) rated by investigators (in person) and by 5 independent video raters of patient videotapes. The secondary endpoints are the change from baseline to day 28 for the TETRAS Activity of Daily Living schedule (TETRAS-ADL) and for the Kinesia One score. There are several exploratory endpoints. The change from baseline to day 15 and 28 for Total TETRAS (PS plus ADL) score and for Kinesia One will be measured. The change from baseline to day 15 for the TETRAS-PS (investigators and independent video rater) and Kinesia One Score will be evaluated. Treatment success at the end of therapy will be measured by Patient Global Impression of Change (PGIC), Clinical Global Impression of Improvement (CGI-I), Goal Attainment Scaling (GAS), and Quality of Life in Essential Tremor Questionnaire (QUEST).

There are two exploratory endpoints for the T-CALM digital substudy. Kinesia 360 will measure the change in tremor amplitude from baseline to days 15 and 28. The iMotor test will evaluate five simple motor functions including digital spirography from baseline to days 15 and 28.

### ET Performance Scales and Biometric Monitoring Devices

Several performance scales and an objective biometric monitoring device will be utilized in the main T-CALM study. These tools will generate relevant and convergent efficacy data that may more accurately define the response of patients to ET therapies. All site investigators will receive training on use of performance scales and biometric monitoring devices to generate high quality and replicable ET scoring data.

The Essential Tremor Rating Assessment Scale (TETRAS) was recently published (53) and is composed of a 9-item performance subscale and a 12-item activities of daily living (ADL) subscale. These subscales provide a rapid clinical evaluation (< 10 min) of ET using pen and paper methodology. The performance subscale measures tremor amplitude (severity) in the head, face, voice, limbs and trunk as well as functional tests including handwriting, drawing a spiral and holding a pen over a dot on a 5-point rating scale where 0 represents no tremor and 4 indicates severe tremor. The sum of the individual scores generates an overall performance subscale score from 0 to 64. The TETRAS-PS will be scored by both investigators at the clinical site and by 5 independent video raters. The scores will be statistically analyzed as the change from baseline to day 28 and serve as the primary efficacy endpoint. The most optimal rating methodology (investigator vs. independent video rated TETRAS-PS) will be selected for late-stage development. The performance subscale data will also be used for exploratory analysis of efficacy on day 15. The ADL subscale evaluates activities of daily living such as speaking, eating, drinking, dressing, personal hygiene, writing, and carrying items. The patient will score each item from 0 (normal activity) to 4 (severe abnormality). The overall score ranges from 0 to 48 and will be analyzed as the change from baseline to day 28 as a secondary efficacy endpoint. The total TETRAS score (sum of the performance subscale and ADL) on the change from baseline to day 15 and 28 will be evaluated as an exploratory endpoint.

Kinesia One platform will be deployed in T-CALM as a digital marker of tremor severity. Kinesia One is FDA cleared for monitoring Parkinson's motor symptom severity with only limited data on assessment of tremor in ET patients. The algorithmically derived score has been developed primarily in Parkinson's disease algorithms and has limited validation in ET (54). The Kinesia One platform integrates accelerometers and gyroscopes to capture kinetic movement disorders (55, 56). The Kinesia One device will be placed on the index finger of each ET patient and worn in the clinic after completion of the TETRAS-PS. Four tasks will be performed by the patient on the left and right sides to assess resting, postural, kinetic and lateral wing beating tremor. The Kinesia One score change in score from baseline to day 28 will be evaluated as a secondary efficacy endpoint. The Kinesia One data will also be utilized for exploratory analysis of efficacy on the change from baseline to day 15 for accelerometry score and for change from baseline on amplitude measures for days 15 and 28. Consistent finger sensor placement and consistent task execution are critical factors for valid Kinesia One scores.

Due to the deleterious effects of ET on daily activities and well-being, several quality of life assessments will be conducted to more accurately assess the patient's perception of the disorder and the effects of pharmacotherapy from baseline to the conclusion of treatment (day 28). Each of these questionnaires and scales requires substantial input from the ET patient and the data will be used to address exploratory efficacy endpoints.

QUEST (57) will be used to evaluate the consequences of ET on daily life of ET patients from baseline to day 28. The questionnaire contains 30 items that involve 5 subscales (physical, psychosocial, communication, hobbies/leisure, and work/finance) and a total score. There are also three additional items that pertain to sexual capability, satisfaction with tremor control, and side effects of pharmacotherapy. If the treatment program for ET is beneficial whether symptomatic or curative, patients will likely respond in a positive manner to QUEST. The QUEST includes questions that are not expected to change within a 28-day timeframe. However, some items such as the satisfaction with tremor control may generate insightful data.

CGI will generate a clinician's perception of a patient's functioning prior to and after study medication (58). The CGI overall score considers patient history, psychosocial situations, symptoms and behavior with respect to the ability to function. The CGI-Improvement (CGI-I) will involve a single 7-point rating of total improvement or change from baseline CGI-Severity (CGI-S). The clinician rater will select one response (from 1 = very much improved to 7 = very much worse) based upon the question “Compared to your patient's condition at the onset of treatment, how much has your patient changed?”

PGIC will quantify a patient's impression of improvement or decline over time with respect to ET treatment (58). The patient will use the PGIC scale to assess current health status vs. initiation of treatment and calculate the difference. The 7-point scale will ask the question “With respect to your ET, how would you describe yourself now compared to when you started taking the study drug?” The patient will reply with one of seven answers from very much worse to very much improved.

GAS (59) will require interaction between the physician and ET patient for development of a written set of individual patient-desired goals to track progress of treatment. At baseline, each patient establishes 3 individual health goals and rates each goal as fairly important = 1, very important = 2, or extremely important = 3. The clinician will rate the degree of difficulty for each goal as probable = 1, possible = 2, or doubtful = 3. During the study, progress will be scored on a 5-point scale from worse than baseline = −2 to best anticipated outcome = +2.

Two additional biometric monitoring tools will be employed in the T-CALM digital substudy to explore their ability to measure changes in motor function of ET patients. The data will be used for exploratory efficacy endpoints.

The Kinesia 360 (Great Lakes Neuro Technologies, Cleveland, OH, USA) (55, 56, 60) is a home monitoring system that utilizes wrist and ankle sensors to objectively and continuously tabulate motion data The Kinesia 360 kit contains a smartphone with the installed Kinesia 360 application, two wearable sensors and charging equipment. The sensors capture 3-dimensional linear acceleration and angular velocity from the wrist and ankle of each ET patient throughout each day with the use of integrated accelerometers and gyroscopes. At the end of each day, the motion data are uploaded from the smartphone to a central server. The data are processed to detail the occurrence and severity of tremor as well as the patient's level of daily activity.

The iMotor (Apptomics, Inc., Wellesley Hills, MA, USA) (61) is a tablet-based application that objectively measures motor function in patients with abnormal movement. The iMotor test will only be conducted during scheduled visits. Each ET patient will be required to conduct 5 simple tasks (finger tapping, hand tapping, hand pronation and supination, reaction to a mild stimulus, and a spiral drawing with a digital stylus) on a tablet. Each task will have a time limit of 30 s and will be done twice (once with each hand).

### Adverse Events

All treatment-emergent adverse events will be coded into the Medical Dictionary for Regulatory Activities (MedDRA) version 20 with system organ classes and preferred terms and displayed in frequency tables by treatment group. Adverse events will be characterized by maximum severity, drug-related adverse events, serious adverse events and adverse events leading to discontinuation of study.

Other safety assessments will include physical examination, neurological examination, vital signs, clinical laboratory tests (hematology, chemistry, and urinalysis), urine drug screen, pregnancy tests, electrocardiogram (ECG), Columbia Suicide Severity Rating Scale (C-SSRS) (62), Epworth Sleepiness Scale (ESS) (63), and University of Miami Parkinson's Disease Hallucinations Questionnaire (UM-PDHQ) (64).

### Statistical Analysis Plan

All statistical analyses will be performed with the SAS system, version 9.4 or higher. A sample size of 43 patients per treatment group has at least 90% power to detect at least a 5.5-point difference between CX-8998 and placebo for the primary endpoint of the change from baseline to Day 28 on the TETRAS-PS score with a standard deviation of 7.5 and alpha = 0.05. This calculation is based on the Wilcoxon-Mann-Whitney test for 2 independent means and assumed normal distributions for each treatment group with a common, but unconfirmed, standard deviation. Approximately 106 patients are planned for enrollment to insure 86 patients are available for inclusion in the efficacy analyses. Five analysis sets will be employed. The Intent to Treat (ITT) analysis set contains all randomized patients and will be used for patient disposition and demographics. The Safety Analysis Set (SAS) has all randomized patients who receive at least 1 dose of study drug. The Full Analysis Set (FAS) includes all patients who receive at least 1 dose of study drug and have both baseline and at least 1 postbaseline efficacy assessment. FAS will be utilized for all efficacy assessments. The Per Protocol Analysis Set (PPS) includes all patients in the FAS with no major protocol deviations. The PPS will be used as a backup analysis for primary and secondary efficacy endpoints. The Day 28 Completers Analysis Set will be composed of all FAS patients who complete the treatment period. This data set will be used as a backup analysis for primary and secondary efficacy endpoints.

The primary efficacy endpoint will be analyzed with the FAS and analysis of covariance (ANCOVA) model, with fixed effects for treatment, anti-tremor medication use, site type and baseline TETRAS-PS score. Testing will be performed with least square (LS) means from the ANCOVA model and a 2-sided test at the alpha = 0.05 level of significance. If the data indicate a departure from the normal distribution, a corresponding rank test will be performed. Multiple imputation will be used to estimate missing data for patients who are missing a TETRAS-PS score on Day 28. Secondary and exploratory efficacy endpoints will be similarly analyzed.

All TEAEs will be coded into MedDRA system organ classes as described above. Descriptive statistics (number, mean, standard deviation, median, minimum and maximum) will be used to summarize observed and change from baseline laboratory, vital sign and ECG data.

Since the T-CALM digital substudy is exploratory, there will not be a formal sample size determination. It is proposed that at least 30 patients will be randomized to CX-8998 or placebo.

### Data Management

Data quality management and monitoring of the trial will be conducted by the sponsor and its designated Contract Research Organization. Substantial protocol amendments will be submitted by the sponsor to regulatory authorities and IRB for approval. Protocol deviations will be documented by the investigator and reported to regulatory authorities and IRB. The sponsor or its designee may conduct audits to insure the study is being conducted in compliance with the protocol, standard operating procedures, GCP and regulatory requirements. The sponsor's study safety representative and a separate independent medically qualified and clinical trials experienced safety physician will monitor aggregate study level safety and tolerability on a recurring basis. An extensive 8-point safety monitoring and risk mitigation plan for adverse events will be used with specific measures to minimize risks to enrolled patients. After completion of Visit 4 by about 75% of study participants, the sponsor may convene an independent external data monitoring committee to review unblinded efficacy data in collaboration with the unblinded study statistician and to provide a recommendation about completion, resizing or termination of the study. The data monitoring committee may request a meeting with the independent safety monitor to discuss safety/tolerability findings in support of its recommendation to the sponsor.

### Informed Consent

The principal investigator (or an appropriate designee) will be responsible for ensuring that each potential study subject is given full and adequate oral and written explanations of the aims, methods, anticipated benefits and potential risks of the study. Signed, written informed consent will be required of each study participant prior to initiation of any procedure.

## Discussion of Anticipated Results and Limitations

The T-CALM main study and substudy are designed to demonstrate the efficacy of CX-8998, a selective TTCC modulator, for treatment of moderate to severe ET inadequately treated with available standard of care approaches. This phase 2, proof of concept, well-powered, multicenter, prospective, randomized, double-blind, placebo-controlled, parallel-group study utilizes physician rating scales, patient-focused questionnaires and functional scales and digital motor function measurements to generate clinically meaningful and congruent efficacy data. Patient perception of the debilitating aspects of ET and the potential benefits of CX-8998 for daily activities and quality of life will be key findings of the study.

It is important to point out that T-CALM is designed as a rigorous, parallel-group study to generate robust efficacy data, reduce dropouts, minimize time of patient participation and maintain double-blind. A crossover design was considered to minimize sample size but rejected due to possible carryover effects between treatments that may compromise the efficacy data (1, 31). Due to limited understanding of the causes and pathophysiology of ET and uncertain diagnosis of the disorder (1), eligibility criteria for the T-CALM study were carefully established and will be enforced to ensure that patients with moderate to severe ET enter the trial. Critical inclusion criteria were diagnosis of definite or probable bilateral ET as defined by the Tremor Investigational Group, tremor severity score of at least 2 in at least one upper extremity on at least one of the three maneuvers on the TETRAS scale and total TETRAS performance scale score of at least 15. Key exclusion criteria were direct or indirect trauma to the nervous system within 3 months preceding the onset of tremor, history or clinical evidence of psychogenic tremor origin and known history of other medical or neurological conditions that may cause or explain patient's tremor, including but not limited to Parkinson's disease, dystonia, cerebellar disease (other than ET), traumatic brain injury, etc. Prior surgical intervention for ET was also excluded.

Since there has been a limited number of well-powered, randomized, controlled, late-stage, clinical trials to evaluate ET pharmacotherapies, some of the methodologies for efficacy endpoints are established and validated whereas others are under development. The TETRAS-PS was selected as the semiquantitative method to generate data for the primary endpoint (reduction of tremor severity by CX-8998) of the T-CALM study. TETRAS has several advantages and known limitations as an efficacy measurement tool for ET. This scale is scientifically validated and clinically grounded, conducted expediently (10 min), accurately and comprehensively measures severe upper limb tremor amplitude as well as tremor of head, face, voice, and lower limb, shows strong reliability among raters, lacks the ceiling effect of prior scales and correlates with ADL and motor function measurements (10, 11). The use of independent video rating is hypothesized to reduce investigator bias, placebo effect and variability. The main disadvantages of TETRAS are limited exposure as the primary endpoint in trials of investigational drugs and the inability to rate rest tremor (a rare occurrence in ET patients), provide a comprehensive neurological assessment, evaluate small changes, generate interval data, or evaluate additional motor and non-motor symptoms of ET such as ataxia, gait abnormalities, anxiety, and depression (11, 65). Since TETRAS will be scored by independent video raters on a videotape of each patient and by individual investigators on each patient in live three-dimensional observation, it will be interesting to determine the level of correlation and select the appropriate methodology for future clinical trials. The degree of correlation will also depend on inter-rater reliability and quality of the video, especially for head, trunk, and lower limbs (known issues in the original scale publication). The other frequently used scale for evaluation of tremor is the Fahn-Tolosa-Marin (FTM) rating scale (10). Although the tremor data of this scale generally correlate with TETRAS, FTM will not be used in the T-CALM study due to its lengthy and complicated administration process and known ceiling effect (10). The TETRAS-ADL subscale will be used as the semiquantitative tool for a secondary endpoint to assess the effects of CX-8998 on the daily activities of ET patients (11). This validated patient-reported scale is an indicator of everyday life and should support the data of the TETRAS performance subscale. A disadvantage of the ADL and other scales of patient reported outcomes is the challenge to achieve significance in a short duration (28 day) study. The TETRAS-PS and -ADL scores will be combined to allow assessment of the total TETRAS score on efficacy of CX-8998.

The Kinesia One Accelerometer is a validated device for Parkinson's Disease but has limited data for ET (54). This device will be used to perform biometric assessment of reduction of tremor amplitude by CX-8998 in support of secondary and exploratory endpoints. The advantages of this device are objective and precise transducer quantitation of tremor amplitude through combined accelerometers and gyroscopes, generation of interval data and possible correlation with the TETRAS-PS. Until additional data are available, clinical relevance for ET appears to be the main limitation. In addition, placement of the device and exact performance of the tasks may contribute to additional variability of this measure.

Four semiquantitative scales will be assessed through exploratory endpoints to determine treatment success of CX-8998 as perceived by the ET patient through ability to perform daily functions, achievement of specific goals and quality of life. Date generated by PGIC, CGI-I, GAS, and QUEST will be used to enhance support for the beneficial effects for CX-8998 on the physical and functional disabilities inflicted by ET. Although the main limitation will be the subjective nature of the data, this may be mitigated by consistency of the data across the four scales and the relevance of these measures to patients.

Two newly developed, digital platforms (Kinesia 360 and iMotor) that are designed to objectively and quantify motor function in patients with movement disorders will be evaluated through exploratory endpoints in the T-CALM substudy. The obvious advantages of Kinesia 360 will be the continuous capture of quantified motor function during daily activity and the times at which ET motor dysfunction and the level of severity are detected. The Kinesia 360 data will provide objective quantification of the ameliorative effects of CX-8998 on tremor amplitude in ET patients based on integrated accelerometers and gyroscopes. The iMotor is a digital platform developed to monitor and objectively measure functional motor tasks (finger tapping, hand tapping, draw an Archimedes spiral with a digital stylus) in patients with impaired movement. The main attributes of the iMotor technology are the short time interval (30 s) for each task and the precision of the data. The iMotor will generate accurate, quantitative data to substantiate improvement in motor function of individual tasks by CX-8998 in ET patients. Although they may lack the objective nature of quantifying tremor via amplitude-based tasks rated by clinicians or through accelerometry-based devices, the main advantage of Archimedes spirals is that they help to document kinetic tremor during a task reflective of activities of daily living. Drawing of spirals has been an integral part of the routine examination of tremor patients and was integrated into clinical rating scales (66). While graphic evidence of tremor activity is also evaluated clinically by examining writing or drawn spirals as part of the TETRAS per, these are still interpreted subjectively and are not easily standardized across subjects. Thus, the objective and quantifiable data analysis afforded by digital assessment of tremor can be an important tool in research and certain clinical settings (67).

Overall, the goal of T-CALM is to generate robust safety and efficacy data to support a go, no-go decision for further development of the selective TTCC modulator CX-8998 as a treatment for ET. It is also anticipated that the design of T-CALM and use of clinically relevant and convergent efficacy endpoints will guide development of future clinical studies of novel ET pharmacotherapies.

## Contribution to the Field

T-CALM was designed to be adequately powered and enable decision making for further development of CX-8998 for inadequately treated ET. Eligibility criteria were carefully defined to insure selection of ET patients with specific disease requirements. Clinically meaningful and convergent clinician-measured and patient-reported outcomes with validated performance scales, objective biometric tools and quality of life questionnaires are key endpoints to demonstrate changes in tremor severity. It is anticipated that the T-CALM trial will demonstrate that CX-8998 reduces tremor severity on the basis of several clinically relevant and confluent efficacy endpoints and a favorable safety and tolerability profile. If beneficial efficacy and safety data for CX-8998 are evident in the T-CALM trial, further clinical development of this drug as a novel, promising treatment for moderate to severe ET will be warranted. The unique, comprehensive design of T-CALM will likely provide meaningful guidelines for future clinical trials of novel ET pharmacotherapies.

## Data Availability

No datasets were generated or analyzed for this study.

## Ethics Statement

The T-CALM trial will be conducted in accordance with the general ethical principles as stated in the Declaration of Helsinki and in conformance with the International Conference on Harmonization (ICH), Good Clinical Practice (GCP) guidance and applicable US Food and Drug Administration (FDA) requirements regarding IRBs, informed consent, data protection and confidentiality and other statutes or regulations related to the rights and welfare of human subjects participating in biomedical research. The protocol and informed consent document for this study will be reviewed and approved by the institutional review board (IRB) at each participating Investigative site or by a central IRB before the study is initiated at the respective site.

The responsibilities of the sponsor, monitor and investigator are defined in the ICH GCP consolidated guideline, and applicable U.S. regulatory requirements. The investigator is responsible for adhering to the GCP requirements of investigators, for dispensing study drug in accordance with the approved protocol or a signed agreement, and for its secure storage and safe handling throughout the study.

## Author Contributions

SP, ML, SB, and EN have contributed significantly to the concept, strategy and design of the T-CALM protocol. All authors have read, critically revised and approved the final manuscript.

### Conflict of Interest Statement

All authors are full-time employees of Cavion Inc. SP is a stock holder in Cavion Inc.
